# One Year Experience of the Hand Allotransplantation First Performed after Korea Organ Transplantation Act (KOTA) Amendment

**DOI:** 10.1055/a-2059-5570

**Published:** 2023-08-02

**Authors:** Nara Lee, Woo Yeol Baek, Yun Rak Choi, Dong Jin Joo, Won Jai Lee, Jong Won Hong

**Affiliations:** 1 Department of Plastic & Reconstructive Surgery, Yonsei University, College of Medicine, Seoul, Korea; 2 Institute for Human Tissue Restoration, Yonsei University, College of Medicine, Seoul, Korea; 3 Department of Orthopedic Surgery, Yonsei University, College of Medicine, Seoul, Korea; 4 Department of Surgery, Yonsei University, College of Medicine, Seoul, Korea; 5 Department of Transplantation Surgery, Yonsei University, College of Medicine, Seoul, Korea; 6 Transplantation Center, Yonsei University, College of Medicine, Seoul, Korea

**Keywords:** composite tissue allotransplantation, vascularized composite allografting, hand transplantation

## Abstract

The revision of the Korea Organ Transplantation Act (KOTA) in 2018 included hand/arm among the organs that can be transplanted. The first hand transplantation since the revision of KOTA took place in January 2021. A 62-year-old male patient experienced hand amputation on July 13, 2018, by a catapult injury. The patient first visited our institute 3 months after the injury. After serial interviews and an overall evaluation, the patient was registered on the hand transplantation waiting list in January 2020. On January 9, 2021, the patient underwent hand transplantation at the right distal forearm level. The total operation time was 17 hours 15 minutes, and the cold ischemic time was 4 hours 9 minutes. Postoperative immunosuppression was administered based on the protocol used for kidney transplantation. Two acute rejection episodes occurred, on postoperative days 33 and 41. Both rejection episodes were reversible with rescue therapy of a higher tacrolimus trough level, steroid pulse therapy, and topical immunosuppressants. Controlled passive range of motion exercise was started on postoperative day 10. Dynamic splint was applied on postoperative day 18. At 1 year, graft maintenance and functional improvement were satisfactory, and the patient showed a Disabilities of Arm, Shoulder and Hand score of 25.8. We successfully performed the first hand transplantation surgery under the KOTA amendment. It came from the organic and effective cooperation of plastic, orthopaedic, and transplantation departments and we believe it will guarantee the future ongoing success.

## Introduction

The first hand transplantation took place in 1998 in Lyon. 1 Since then, many hand transplantation cases have been reported and reviewed. The surgical process and immunosuppressive regimen have been found both feasible and reliable. 2

Hand/arm vascularized composite allotransplantation (VCA) was registered as a “new health technology” in 2010, which was significant in Korean National Health Insurance. 3 4 However, VCA was not performed in Korea until 2017. Hand/arm was included in the Korea Organ Transplantation Act (KOTA) in 2018 and officially available since then.

The first hand transplantation in Korea was conducted in 2017 by a hand transplantation team from W Hospital and Yeungnam University Medical Center in Daegu, South Korea. After that, KOTA was amended. 5 The amended KOTA began in August 2018 and included hand/arm as a complex tissue of bone, skin, muscles, nerves, and vessels. Hand/arm transplantation is now treated the same as any other organ transplant in Korea. 6 Likewise, hand/arm transplantation is covered by health insurance since 2018, after the amendment of KOTA. The second hand transplantation case, actually the first after KOTA amendment, was performed on January 9, 2021. In this article, we describe the planning and implementation of the surgery, postoperative management, and 1-year experience for hand transplantation.

## Case

### Patient Selection

A 62-year-old male patient had hand amputation on distal forearm level on July 13, 2018, by a catapult injury. The patient first visited our institute for transplant counseling 3 months after the injury. The patient was registered on the transplant waiting list after going through the serial counseling visits and assessments on January 2020.

### Donor Selection

**Fig. 1:**
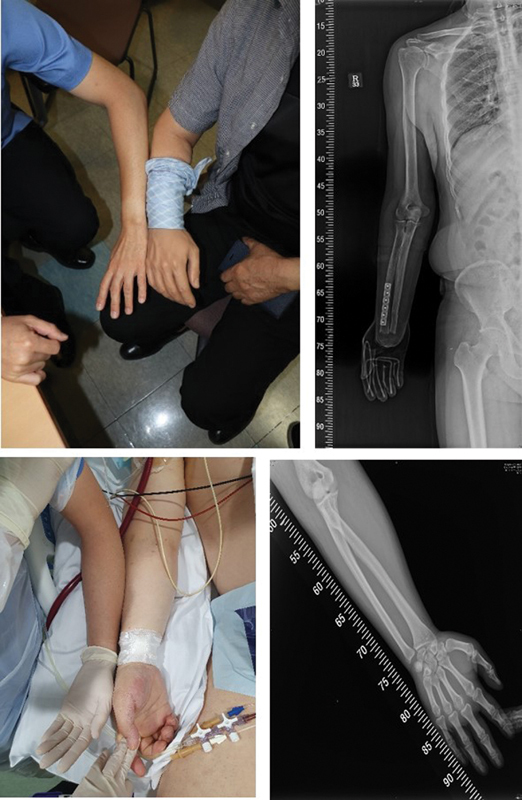
Preoperative photo and scanogram of the recipient (top) and donor (bottom). We compared the recipient and donor arms using the head surgeon's arm (J. W. H.) as a reference. Although we checked arm thickness, color, and length, these were difficult to judge based on figures and photos alone. The scanogram was collected to identify the amputation level and compare the bone thickness and length at that level.

The donor and recipient had ABO- and Rh-matched blood (O Rh positive) and a human leukocyte antigen A (HLA-A), HLA-B and HLA-DR, one-sixth match (donor HLA-A*02:-, HLA-B*44:60, HLA-DR*07:15; recipient HLA-A*02:24, HLA-B*46:55, HLA-DR*8:9). Arm circumference was measured at the proximal one-third of the forearm. The arm circumference of the donor was 34 cm and that of the recipient was 28 cm. Even though the arm circumference was different, the bone thickness could be matched at the bone fixation level. Given all of the objective data, the final suitability of the donor hand was judged “intuitively” using the physician's hand and arm as a reference ( Fig. 1 ).

### Surgical Procedure

#### Donor Procurement

The donor arm was prepared in the lateral extended position so that the liver procurement team could have sufficient space for surgery, and arm and liver procurement were performed at the same time. A nearby operation room was prepared for the reservoir solution washing and table dissection; therefore, there was no delay in the subsequent harvest surgery after arm procurement.

**Fig. 2:**
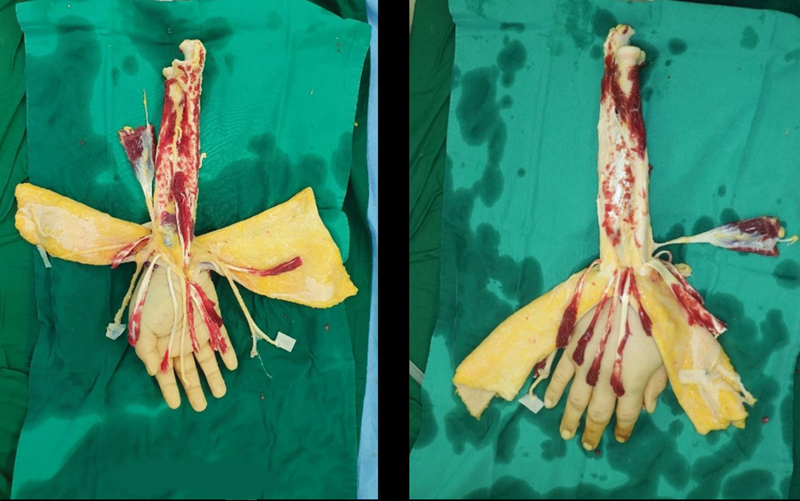
Donor hand table dissection. A total of 2 L of SPS-1 solution was infused through the brachial artery after setting the donor arm on the ice table. A fish-mouth skin flap was designed and elevated to fit the recipient's previous scar. Table dissection was conducted.

**Fig. 3:**
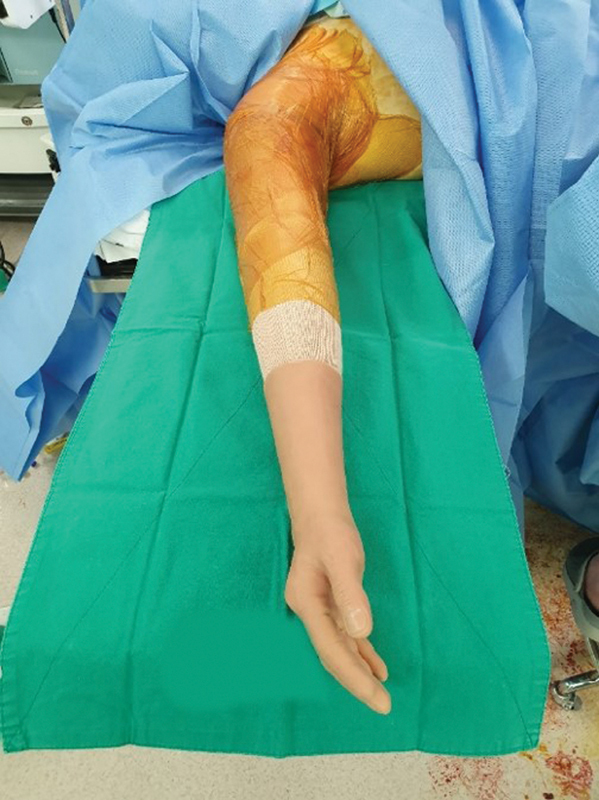
A prosthetic arm was prepared based on the preoperative donor arm data immediately after the brain-death declaration and the decision to donate the hand. The prosthesis was attached to the donor arm at the end of the procurement operation.

Procurement was performed at the upper arm proximal level to get sufficient length for the anastomosis site. The donor arm was then immersed in SPS-1 solution (Static Preservation Solution, UW Solution, Organ Recovery Systems, Belgium), placed in an ice box, and transported to the nearby operation room for dissection. 7 After setting the donor arm on an ice table, dissection was performed ( Fig. 2 ). The donor site was repaired with a purse-string suture. Donor-customized prosthesis was installed on the donor arm before we left the operating room ( Fig. 3 ).

#### Hand Transplantation

**Fig. 4:**
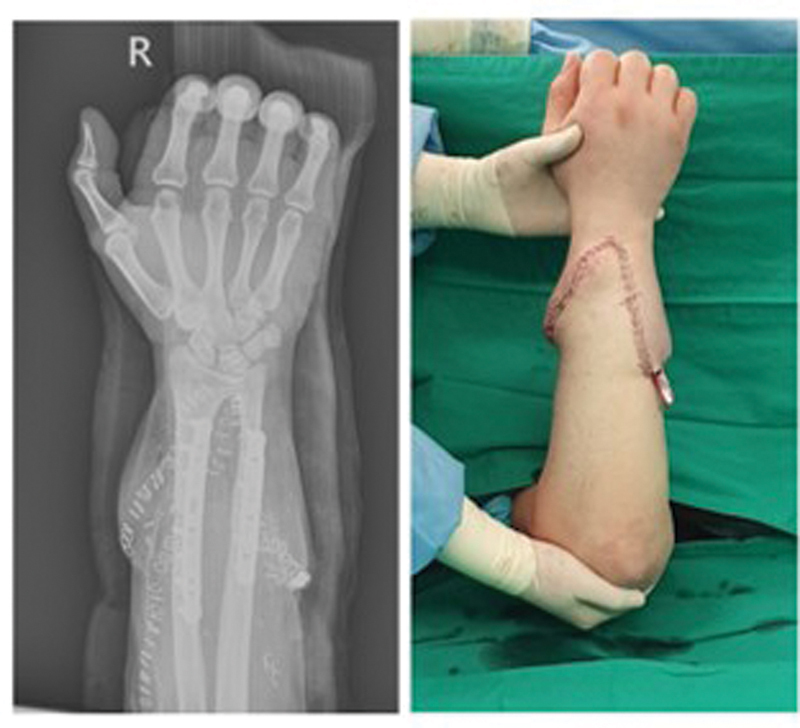
Immediate postoperative gross photo and X-ray. The donor bone was resected 1.7 cm distal to the proximal border of the pronator quadratus muscle to adjust the bone thickness at the bone fixation level. The bone thickness was well matched at the bone fixation level.

Recipient hand preparation was performed simultaneously with donor arm procurement. A fish-mouth design skin incision was made, taking care not to damage the superficial vein and nerves. Bone fixation and extensor tendon repair were performed first. The cephalic vein was temporarily anastomosed first, and then the radial artery was anastomosed. The cold ischemic time was 4 hours 9 minutes. Two venae comitantes were anastomosed before flexor tendon repair. The superficial radial nerve, ulnar nerve, medial nerve, and cutaneous branch of the ulnar nerve were repaired, and anterior interosseous nerve coaptation was performed into the ulnar nerve. The ulnar artery and two venae comitantes were anastomosed. A skin flap was laid without tension, and the margin was well adjusted, as designed. The cephalic vein was trimmed, and reanastomosis was conducted to fit the new arm length. Two silastic drains were indwelled ( Fig. 4 ).

### Postoperative Intensive Care Unit Care and Complications

The patient stayed in the intensive care unit (ICU) for 5 days and received heparin (Hanlim Pharmaceutical Co., South Korea) for 48 hours postoperatively. 7 8 Two pulse oximeter probes were set bilaterally on the patient's index fingers to monitor distal oxygen saturation. Capillary refill was monitored in the fingernails and skin. On postoperative day 2, a subcutaneous hematoma was found. Bedside hematoma evacuation was performed by removing a few stitches.

### Immunosuppressive Regimen

**Fig. 5:**
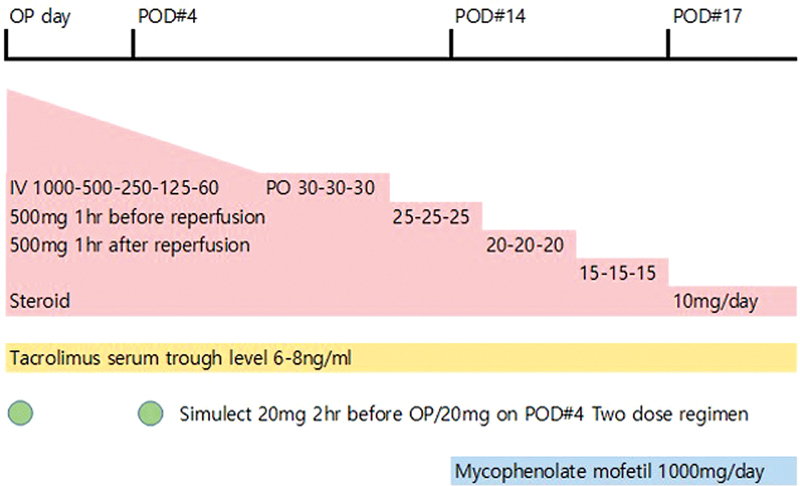
Induction and maintenance immunosuppressive therapy. A triple-drug regimen induction therapy (tacrolimus, steroid, and basiliximab) was administered. Tacrolimus was started on the operation day, with 3 mg given preoperatively and then 4 mg/day given as the initial dose. The target serum trough level of 6–8 ng/mL was tested three times a week. Two 500-mg doses of steroid were intravenously infused 1 hour before and after reperfusion. Basiliximab was administered in two 20-mg doses, 2 hours before the operation and on postoperative day 4. The triple-drug regimen maintenance therapy uses tacrolimus, steroid, and mycophenolate mofetil. The target tacrolimus trough level was 6–8 ng/mL postoperatively. The steroid was tapered to half a dose every day to 30 mg/day. Then, oral steroid tapering reduced the daily dose by 15 mg once every 3 days. The oral dose reached 10 mg per day on postoperative day 17 and was maintained at that level. Mycophenolate mofetil was started on postoperative day 14 with a 1,000-mg daily dose.

The immunosuppressive regimen can be divided into three stages of induction therapy, maintenance therapy, and rescue therapy. We followed the standard immunosuppressive regimen for kidney transplantation ( Fig. 5 ). 9 10

### Acute Rejection Episodes and Rescue Therapy

**Fig. 6:**
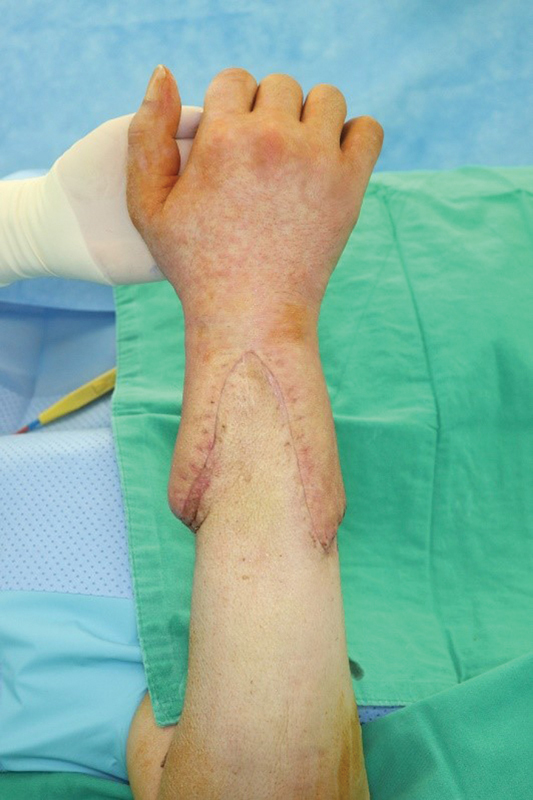
Acute rejection with diffuse swelling and erythema (postoperative day 41). Even though the patient was on high-dose steroid therapy, he experienced a second rejection episode with severe symptoms.

**Fig. 7:**
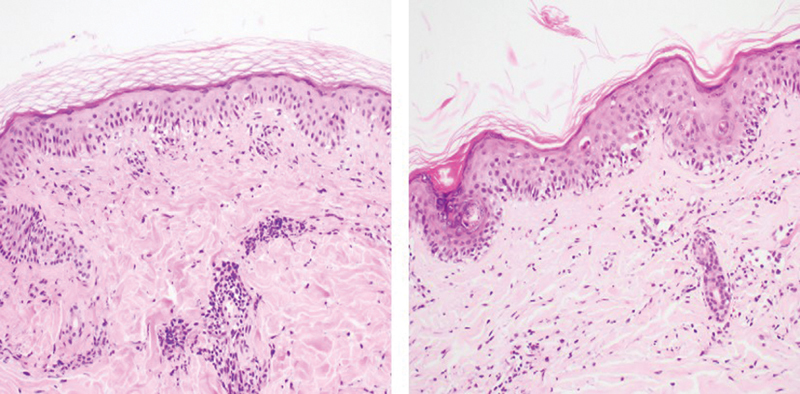
Superficial mild-to-moderate perivenular lymphocytic infiltration with basal vacuolization, Banff I, II (left, postoperative day 33, ×200) with moderate-to-dense inflammation and perivascular lymphocyte infiltration. Focal confluent basal vacuolization with a few necrotizing keratinocytes, Banff II, III (right, postoperative day 41, ×200).

**Fig. 8:**
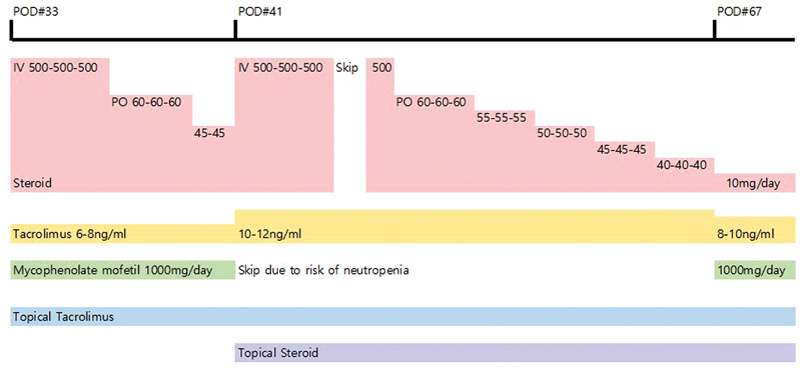
Rescue therapy. The first rejection episode was pathologically confirmed to be Banff grade I. After 3 days of intravenous steroid pulse therapy with 500-mg methylprednisolone per day, it was tapered to an oral dose of 60 mg per day. The daily steroid dose was reduced by 15 mg every 3 days. The tacrolimus serum trough level was maintained at 6–8 ng/mL. Topical tacrolimus was applied. Despite the high-dose steroid infusion and topical tacrolimus therapy, a second acute rejection appeared on postoperative day 41. It was pathologically confirmed to be Banff grade III. We readministered 3 days of intravenous steroid pulse therapy of 500-mg methylprednisolone per day. We took 1 day off from the steroid injections and then administered one more 500-mg methylprednisolone as a tapering dose. Slower steroid t apering was used this time by reducing the oral steroid daily dose by 5 mg every 3 days. It took 27 days to taper the steroid to the maintenance dose of 10 mg per day. The target tacrolimus serum trough level was increased to 10 to 12 ng/mL. A topical steroid was alternated with topical tacrolimus. Because we were concerned about the risk of neutropenia after high-dose steroid application, mycophenolate mofetil was stopped for 27 days.

The patient experienced acute rejection episodes on postoperative days 33 and 41. The first rejection episode was noted during a routine postoperative follow-up visit after hospital discharge and appeared as diffuse swelling and erythema ( Fig. 6 ). A punch biopsy (2 mm) was taken, and mild rejection with Banff grade I was confirmed ( Fig. 7 ). Steroid pulse therapy was promptly administered. After 3 days of intravenous steroid pulse therapy with 500 mg of methylprednisolone per day, it was tapered to an oral dose of 60 mg per day. The daily steroid dose was then reduced by 15 mg every 3 days. At the same time, topical tacrolimus was applied. The tacrolimus serum trough level was maintained at 6 to 8 ng/mL ( Fig. 8 ).

Despite the increased steroid infusion and topical tacrolimus therapy, a second acute rejection occurred on postoperative day 41. Two punch biopsies (3 mm) were taken, and moderate-to-severe rejection with the Banff grade III was confirmed ( Fig. 7 ). After 3 days of intravenous steroid pulse therapy (500 mg per day, intravenously), slower tapering of the steroid was performed this time. The tacrolimus serum trough level was increased to 10 to 12 ng/mL. A topical steroid was additionally applied alternately with topical tacrolimus. To mitigate the risk of neutropenia after high-dose steroid application, mycophenolate mofetil was stopped for 27 days ( Fig. 8 ).

### Revision Surgery

**Fig. 9:**
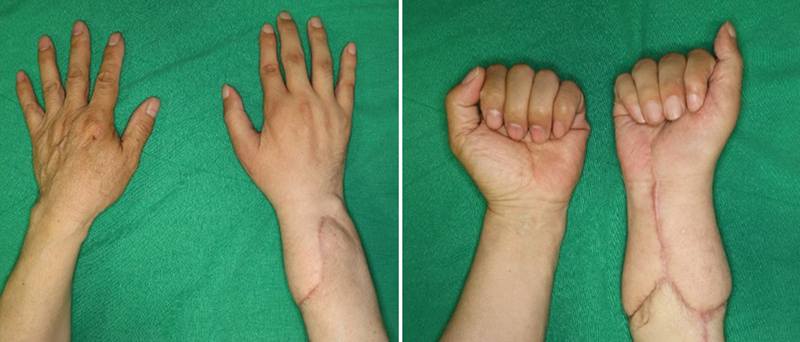
Follow-up at 16 months. Voluntary movement of the distal interphalangeal joint and proximal interphalangeal joint enabled grip motion. Recovery of pinch motion was late due to weak thumb opposition.

At the 1-year follow-up visit, the patient's grip and finger flexion and extension had recovered, but recovery of pinch, thumb abduction, and thumb extension was slow. Tenolysis and median nerve neurolysis with carpal tunnel release was conducted. Simultaneously, a debulking procedure was conducted by excising the recipient-side scar tissue. First metacarpophalangeal joint fusion was performed to enable a reliable pinch motion. After revision surgery, the patient is visiting routine outpatient visits without any specific complications ( Fig. 9 ).

## Discussion

With the KOTA revision in 2018, hand/arm transplantation became available in South Korea. However, the donor should be available in the recipient-enrolled hospital. Sharing “hand organ” is prohibited in South Korea by transplantation law because it is still pioneeric medical part.

Institutional support for hand/arm transplantation in South Korea has some limitations. Transplantable organs other than hand/arm can be transported from one hospital to another, but a patient who is registered on a particular hospital's waiting list for a hand/arm can only receive a hand/arm harvested from a brain-dead donor in that hospital. In other words, patients can face a longer waiting period for transplantation because they cannot receive a hand/arm harvested from a matching brain-dead donor from another hospital. Our patient first visited our outpatient department in 2018, after the KOTA revision, and waited for 1 year between registration on the waiting list and hand/arm transplant surgery.

Although there is disadvantage in requiring harvest and transplantation be performed within the same hospital, it does have the advantage of allowing the transplant team to prepare the transplantation immediately upon donation. In our institution, the plastic surgeon (J. W. H) acts as the director of the whole process and was able to side-by-side compare the donor and recipient arms with his own arm. It was difficult to judge suitability based on data and photos alone, and his hand was deemed to be an appropriate reference hand ( Fig. 1 ). Additionally, after prompt verification of the size, color, and length of the hand/arm, circumference of hand, distal and proximal one-third of forearm, elbow, and distal and proximal one-third of upper arm were measured, with the measurement of bone length and bone thickness of the expected amputation level on radiography.

The transplantation itself was carried out as a cooperative work of the plastic surgery, orthopaedic surgery, and transplant surgery departments. Skin flap design and muscle dissection were conducted by the plastic surgeon, the bones and ligaments were treated by the orthopaedic surgeon, and immunosuppressants were prescribed and administered by the transplant surgeon.

During the operation, nearby operating rooms were available, allowing us to wash and perform table dissection without interfering with other harvest teams or recipient preparation, which was in progress in another operating room at the same time. Just after bone fixation, we repaired all extensor tendons for wrist stability. The cephalic vein was anastomosed first for drainage after arterial anastomosis, and then the radial artery was anastomosed to minimize the ischemic time. Several previous replantation cases had shown that one cephalic vein anastomosis is sufficient for entire arm maintenance. 11 12 The cephalic vein was later trimmed to match the new arm length and reanastomosed.

Acute rejection can occur several times within 1 year after transplant surgery, and physicians should control the immunosuppressant dosage and adjust the maintenance regimen with close monitoring of the patient's response. 13 Our patient had two rejection episodes, one on each of postoperative days 33 and 41. As a rescue therapy, we administered steroid pulse therapy and topical tacrolimus. Intravenous antibiotics were also started because we could not rule out the possibility of a postoperative infection. The rejection episode seemed to be stabilizing after steroid pulse therapy. However, 9 days after the first episode, a second rejection episode was noted. Because the patient had already received a high dose of intravenous steroid 9 days ago, we were concerned about proper treatment. Nonetheless, we decided that we had to restart steroid pulse therapy. At that time, we also increased the tacrolimus serum trough level target with alternative application of a steroid ointment with tacrolimus ointment. Because we were concerned about neutropenia after the repeated high-dose steroid administration, we implemented 1 day off after 3 days of intravenous steroid pulse therapy. For the same reason, we paused administration of mycophenolate mofetil and restarted it after steroid tapering. After the second episode, we tapered the oral dose of steroids more slowly than usual ( Fig. 8 ).

Beginning 2 months after the operation, grip with finger flexion was possible, allowing the patient to hold a steering wheel or change gears. Posttransplantation, he is satisfied that he can adequately use both hands and feel free from that social stress, for example, when handling with only one hand when he was carrying a bag and trying to fold an umbrella at the same time, or when trying to put something out of the bag while carrying it. He was satisfied that he can handle it with both hands and feels free from the social stress.
